# Patterns recovered in phylogenomic analysis of Candida auris and close relatives implicate broad environmental flexibility in Candida/Clavispora clade yeasts

**DOI:** 10.1099/mgen.0.001233

**Published:** 2024-04-17

**Authors:** Kyle Schutz, Tina Melie, Stacey D. Smith, C. Alisha Quandt

**Affiliations:** 1 Department of Ecology and Evolutionary Biology, University of Colorado, Boulder, USA

**Keywords:** *Candida auris*, *Candida haemulonii*, yeast phylogenomics

## Abstract

Fungal pathogens commonly originate from benign or non-pathogenic strains living in the natural environment. The recently emerged human pathogen, *Candida auris,* is one example of a fungus believed to have originated in the environment and recently transitioned into a clinical setting. To date, however, there is limited evidence about the origins of this species in the natural environment and when it began associating with humans. One approach to overcome this gap is to reconstruct phylogenetic relationships between (1) strains isolated from clinical and non-clinical environments and (2) between species known to cause disease in humans and benign environmental saprobes. *C. auris* belongs to the *Candida/Clavispora* clade, a diverse group of 45 yeast species including human pathogens and environmental saprobes. We present a phylogenomic analysis of the *Candida/Clavispora* clade aimed at understanding the ecological breadth and evolutionary relationships between an expanded sample of environmentally and clinically isolated yeasts. To build a robust framework for investigating these relationships, we developed a whole-genome sequence dataset of 108 isolates representing 18 species, including four newly sequenced species and 18 environmentally isolated strains. Our phylogeny, based on 619 orthologous genes, shows environmentally isolated species and strains interspersed with clinically isolated counterparts, suggesting that there have been many transitions between humans and the natural environment in this clade. Our findings highlight the breadth of environments these yeasts inhabit and imply that many clinically isolated yeasts in this clade could just as easily live outside the human body in diverse natural environments and vice versa.

**Impact Statement**

The rapid rise in the number of fungal pathogens over the past few decades has been linked to climate change and an increase in immunocompromised individuals. *Candida auris* is an example of a recently emerged fungal pathogen capable of causing severe disease and large outbreaks in vulnerable patient populations. The evolutionary origins of *C. auris* are poorly understood, however, they are essential to understanding how this yeast interacts with humans and when that interaction started. In this study, we investigated relationships between a sample of environmentally and clinically isolated strains and species in the *Candida*/*Clavispora* clade, a group of 45 yeast species including *C. auris* and environmental saprobes. Our phylogenetic analysis revealed that clinically isolated strains do not comprise a single lineage and are instead intermixed with environmental strains, implying many transitions between humans and other environments. It seems plausible that *C. auris* and related species, often found in harsh environmental conditions such as seawater, already possessed traits that make them capable of acting as human pathogens. Thus, we should be vigilant in monitoring the prevalence of these species, particularly in proximity to at-risk patient populations.

## Data Summary

Workflows and scripts used to generate these data are publicly available on Kyle Schutz’s GitHub https://github.com/kyleschutz/candida_clavispora_workflow. Raw read sequence data were deposited in NCBI’s SRA Database under BioProject PRJNA945431. Genome assemblies, annotations, and trimmed alignments are available at: https://doi.org/10.5281/zenodo.8408925. Individual ML gene trees are available at: https://zenodo.org/record/8411131.

## Introduction

The spectrum of fungal disease in humans is diverse, as are the microevolutionary trajectories that shape how fungi interact with humans. Negative human-fungal interactions can result in a range of disease presentations, from minor skin infections to life-threatening systemic mycoses. These diseases are often triggered by changes in the human body, such as weakened immune systems or chronic illnesses [1,2]. Many well-studied opportunistic fungal pathogens are known to cause disease in immunocompromised or sick humans, such as *Candida albicans*, *Cryptococcus neoformans,* and *Coccidioides immitis* [1]. Over the last 30 years there has been a sharp rise in the number of new fungal species isolated from the human body [2]. In addition to changes in overall human health and our access to medicine, this rise has also been attributed to climate change, where warming ambient temperatures may select for thermotolerant fungal strains (growth above 42 °C) able to overcome the mammalian thermal restriction zone (37 °C in humans) [3]. These hypotheses have generated much interest within medical mycology, given the increase in sicker individuals due to the COVID-19 pandemic [4] and the growing impact of climate change on the emergence of fungal pathogens [5].

The budding yeast, *Candida auris*, has been presented as the first example of a serious human fungal pathogen to have emerged due to climate change [3,6]. In the United States, screening cases of this yeast have tripled from 2020 to 2021, likely due to increased hospitalization rates secondary to the COVID-19 pandemic [4]. To date, however, there is limited evidence about this species’ origin in the natural environment and when it began associating with humans – two important pieces needed to evaluate the impact of climate change on emerging human fungal pathogens. *C. auris* was officially first reported in 2009 when it was isolated clinically from a patient with otitis media in Japan [7]. The earliest clinical isolate of *C. auris*, however, dates to 1996, when it was misidentified as *Candida haemulonii* in Korea [8]. Given that fungal disease reporting before the 1990s is sparse, it is challenging to determine exactly how long *C. auris* has been associating with humans. Nevertheless, the clinical emergence of *C. auris* is relatively recent when compared to other human-associated fungi in subphylum *Saccharomycotina*, such as *C. albicans* which was first described from a clinical source in 1839 and has likely long been associating with humans [9]. Phylogenetic analysis and Bayesian molecular clock dating of clinical *C. auris* strains have attempted to date the beginning of human associations. One study of clinical strains suggests that *C. auris* diverged from their most recent common ancestor as recently as 360 years ago [10]. Analysis of population structure shows that clinical *C. auris* strains group into five, genetically distinct clades with divergences as recent as ~35 years ago [10]. Although these sub-clades (I-V) have been given geographic labels to help with outbreak tracing, it is unclear if the sub-clade strains arose from sources in these geographic locations leading to multiple outbreaks across the globe, or if there was a point-source reservoir that has been exacerbated by global travel [10,12].

Broadly, fungi must overcome many physiological challenges to survive the human body, including growth above 37 °C, while simultaneously evading the human immune response [12]. How *C. auris* interacts with humans is unique when compared to distantly related, opportunistic pathogens such as *C. albicans* and *C. glabrata*. Whereas *C. albicans* and *C. glabrata* typically colonize the gastrointestinal tract [13], *C. auris* commonly colonizes the salty and warm skin compartment, which allows yeast cells to easily shed and move between patients and their direct environment [14,16]. Colonizing this niche in the human body likely explains *C. auris’* propensity for large outbreaks in clinical settings [4], where sick individuals are housed in close proximity, often sharing many medical providers and equipment [17]. In this setting, yeast cells are then able to enter body through indwelling devices, such as catheters, resulting in serious systemic infections [4]. *C. auris’* success in the human body has been attributed to its ability to consistently grow above 42 °C *in vitro*, whereas other close relatives grow weakly above 35 °C in the same conditions [18]. This trait, coupled with *in vitro* growth in highly saline conditions (10 % wt/vol) in culture has led many to question whether *C. auris* is living in the natural environment [17].

Until recently, *C. auris* had not been isolated from the natural environment, making it unclear as to whether there was an ecological niche for the yeast outside the human body and occasional piece of hospital equipment. Given its thermo- and halotolerant phenotype, it had been hypothesized that clinical *C. auris* strains could have closely related environmental strains living in humid, salty environments, such as tropical salt marshes [3]. This assumption underlies the ‘Global Warming’ hypothesis, where warming ambient temperatures may have selected for strains able to overcome the 37 °C mammalian restriction zone, which through the passage through an intermediate non-human host, gave rise to lineages able to survive the human body [3,12]. In 2021, *C. auris* was isolated from salt marshes and beaches in the Andaman and Nicobar Islands in Southeast Asia [6] and in 2022, from seawater in Columbia [19], consistent with a niche in marine or brackish-water environments. Most recently, *C. auris* has also been detected in wastewater surveillance during a hospital outbreak in Nevada [20] and has been identified in public metagenome databases, including wastewater from New Delhi [21]. Although these studies demonstrate that *C. auris* cells can survive outside the human body in harsh environmental conditions, such as seawater and wastewater, it is unclear if these environmentally isolated strains represent distinct non-pathogenic lineages or if they are strains that have seeded the environment from human sources, such as hospital and human waste.

Although emerging human fungal pathogens can originate in different ways, they commonly have evolutionary histories in benign or non-pathogenic species living in the natural environment [22]. It is also possible that incidentally surviving an immunocompromised human body could enhance fungal fitness and become part of the yeast’s natural lifestyle [23]. Moreover, these yeasts could have transitioned between humans and the natural environment multiple times, and that global surveillance of clinical isolates only represents the tip of *C. auris’* diversity. Considering the shared evolutionary histories of other closely related yeasts with similar lifestyles, such as *Candida haemulonii* and *Candida vulturna,* is one approach to better understanding *C. auris’* success in humans. Both *C. haemulonii* and *C. vulturna* have been increasingly isolated from human sources, raising concerns about the pathogenic potential and transmission dynamics of these otherwise benign, environmental yeasts [18,24, 25]. *C. auris* belongs to the *Candida/Clavispora* clade (*Metschnikowiaceae*), a group of 45 yeast species isolated from diverse sources, including human, flower nectar, insects, and seawater [3,26,31]. The exact placement of *C. auris* within the clade (and thus the identity of its closest relatives) remains uncertain as previous phylogenetic studies have estimated *C. auris* sharing a most recent common ancestor with *Candida ruelliae* [30] or with *Candida heveicola* [3]. Moreover, while whole genome data has been available for *C. auris* for several years [32], we lack such genomic data for close relatives, precluding more robust phylogenetic inference within the *Candida/Clavispora* clade [33].

Along with *C. auris*, five of the 45 described species of the *Candida/Clavispora* clade are consistently isolated from human sources and are known to cause disease (*C. haemulonii*, *Candida pseudohaemulonii*, *Candida duobushaemulonii*, *Candida vulturna,* and *Clavispora lusitaniae*) [34]. All these species (apart from *C. pseudohaemulonii*) have also been recovered from environments outside of the human body. For example, *C. haemulonii* was first isolated in Florida in 1962 from the gut of a blue-striped grunt (*Haemulon sciurus*), and it has since been isolated from seawater in Portugal and other marine sources [35]. *C. haemulonii* was first identified clinically in 1984 and has since increased in prevalence across the globe [36,37]. Similarly, *C. duobushaemulonii* was first isolated from insect frass in Germany [31] and has since been detected in clinical samples, ranging from superficial to invasive [24]. The most recent clinically emerging member of this clade is *C. vulturna*, which was first isolated from flowers in the Philippines and has recently been isolated from blood and other human sources [25,30]. The wide range of environments from which members of the *Candida/Clavispora* clade have been recovered suggests that these yeasts can survive in broad ecological niches and are likely metabolically diverse. This makes it challenging to discern how frequently these yeasts encounter susceptible human hosts and if these interactions, over time, select for specialist traits associated with long-term success in the human body.

Chronicling the evolutionary relationships between strains is a clear first step in assessing transitions between humans and the environment across species in the *Candida/Clavispora* clade [38]. Using a model-based phylogenomic approach provides a robust statistical framework for inferring patterns of evolution, allowing for meaningful comparison of relationships between less divergent species, including genomes from more closely related species as well as several strains per species [38]. While earlier research relied on marker genes for estimating relationships among fungal pathogens and their relatives [3,29,31], studies are increasingly moving to the use of whole genome sequence data [39], resulting in greater clarity and confidence in phylogenetic estimates. Phylogenomic studies often leverage single-copy orthologous coding regions, which typically number in the hundreds in fungal lineages [26] and can provide resolution of both deep and shallow relationships [40].

In this study, we present a phylogenomic analysis of the *Candida/Clavispora* clade based on 619 single-copy orthologs aimed at testing for patterns implicated by differing emergence hypotheses with an expanded sample of strains and species isolated from non-human sources. Assuming complex adaptations are required for survival in the human body, we might expect clinical samples isolated from blood to form a single clade, consistent with one major transition between environments. However, given the diverse, non-human isolation sources of some species and strains in this complex, it is possible that humans have been colonized multiple times through interactions in the natural environment, such as seawater. If this were the case, we would expect to see environmentally isolated strains interspersed with clinically isolated strains on the phylogeny, consistent with the notion that these yeasts are moving back and forth between humans and the environment. To test these hypotheses, we developed a whole-genome sequence dataset of 108 isolates representing 18 species, including four newly sequenced species and 18 environmentally isolated strains to build a robust framework for investigating these relationships. Our findings provide a foundation that chronicles the existing diversity and evolutionary relationships for a clade of yeasts increasingly associated with serious human disease.

## Methods

### Taxon sampling

A total of 108 isolates representing 18 different species were sampled, including four species newly sequenced in this study. A maximum of 20 isolates were randomly selected for species with more than 20 isolates available. These isolates were mostly obtained from clinical specimens (66.6 %), followed by the abiotic environment (15.0 %), plants (11.0 %), animals (5.5 %), and insects (0.9 %) (S1 Appendix). Eight *Candida* spp. isolates (*C. haemulonii* CBS 5150, *C. heveicola* CBS 10701, *C. heveicola* CBS 7249, *C. dosseyi* CBS 10313, *C. duobushaemulonii* CBS 9754, *C. ruelliae* CBS 10815, *C. mogii* CBS 2032 and *C. hainanensis* CBS 10696) were obtained as lyophilized cultures from the Westerdijk Fungal Diversity Institute (https://wi.knaw.nl/) for whole genome sequencing (Table 1). *Saccharomyces cerevisiae* and *Metschnikowia bicuspidate* were selected as outgroups for our phylogeny, based on their phylogenetic distance from the *Candida/Clavispora* clade [26]. All trees were rooted on *S. cerevisiae*.

**Table 1.** Description of whole genomes sequenced from isolates in this study

Species	Strain	Collection country	Substrate	Collect date	CDS region	BUSCO score (%)	GC %	N50	Assembly size (Mb)
*C. dosseyi* [64]	CBS 10313	USA	*Ululodes macleayanus*	2005	6430	99.3	53	308944	15.5
*C. duobushaemulonii* [31]	CBS 9754	Germany	*Pyrrhocois apterus*	2012	5299	98.8	49.39	352276	15.2
*C. haemulonii* [35]	CBS 5150	Portugal	Seawater	2012	5265	98.8	47.62	268833	15.2
*C. hainanensis* [63]	CBS 10696	China	Flower - *Magnoliaceae*	2008	5195	99	52	393541	12.6
*C. heveicola* [64]	CBS 7249	China	Rubber Tree Sap	2008	8246	99.2	49.22	550567	15.6
*C. heveicola* [64]	CBS 10701	China	Rubber Tree Sap	2008	5292	99.1	47	268080	22.3
*C. mogii* [65]	CBS 2032	Japan	Fucho-Miso	1967	4737	98.8	46	112524	12.9
*C. ruelliae* [66]	CBS 10815	India	Flower - *Ruelliacae*	2012	5210	98.7	46	545101	14.2

### DNA extraction and whole genome sequencing

The eight lyophilized isolates were revived on Potato Dextrose Agar using aseptic technique. From each plate, a colony was randomly selected and used to inoculate 10 ml of Sabouraud Dextrose Broth in a 50 ml conical Falcon tube. The tubes were incubated at 25 °C in a shaking incubator at 4000 r.p.m. for 72 h. After incubation, the cultures were centrifuged, and pellets were harvested. Cells were resuspended in a SDS buffer solution and vortexed. The contents were added to Qiagen PowerBead tubes with 0.5 mm glass beads and vortexed for 5 min. The tubes were then heat shocked at 60 °C for 60 s. DNA was extracted using a Qiagen Blood and Tissue Extraction kit. The sample was eluted with warm sterile water rather than the supplied elution buffer. The ITS region was amplified (ITS1-F and ITS4) from the extracted DNA of each isolate and sequenced to confirm species identification and extraction quality. Library preparation was conducted at the University of Colorado Anschutz UCDAMC Genomics Core and sequenced on the Illumina NovaSeq 6000. Raw sequence read data are available in NCBI under BioProject PRJNA945431.

### Genome assembly and annotation

Workflows and scripts used to process these data are publicly available on GitHub: https://github.com/kyleschutz/candida_clavispora_workflow. Raw sequence reads from 82 isolates were *de novo* assembled in this study. Reads from 74 isolates were downloaded from the NCBI Sequence Read Archive using SRA Toolkit’s fasterq-dump. Reads from the eight purchased isolates were also *de novo* assembled. Read quality was assessed with FastQC to inform read trimming parameters [41]. Raw reads were trimmed using Trimmomatic version 0.39 [42] with a sliding window cutoff of 4 : 10 and *de novo* assembled with SPAdes v. 3.15.2 using default settings [43]. Genome assemblies for 26 isolates without raw sequence data available were retrieved from NCBI’s GenBank – accession numbers are available in the S1 Appendix. Assembly statistics were evaluated for all assemblies using Assemblathon 2 [44]. Given that we used a combination of *de novo* assemblies (*n*=82) and available assemblies from GenBank (*n*=26), annotation was performed for all assemblies using Funannotate version 1.8.9 to control for quality and standardize for downstream analyses [45]. Although many of these yeasts encode CUG from leucine to serine, proteins were annotated using *S. cerevisiae* genetic code. Assembly and annotation data are available for download: https://doi.org/10.5281/zenodo.8408925.

To assess the quality of the assemblies and their subsequent annotations using our pipeline, our assembly of *Candida haemulonii* B11899 was compared to a high-quality assembled genome of *Candida haemulonii* B11899 using a custom database query with blastn [46]. This validation test showed 100 % identity between coding regions in the two assemblies. Assembly completeness was assessed using BUSCO version 3 using the Saccharomycetes OrthoDB v. 10 database [47] for all strains (S1 Appendix, available in the online version of this article).

### Identifying orthologous gene clusters

From the 110 input genomes, nucleotide coding sequence data (CDS) were translated to amino acid sequences using EMBOSS v. 6.6.0 [48]. In parallel, CDS regions from all isolates were concatenated to serve as a reference for downstream translation back to nucleotide sequences. In total, 619 single-copy orthologous genes were detected from translated CDS regions using Proteinortho6 and extracted using the grab_proteins.pl script [49]. Each gene cluster was individually aligned with MAFFT v. 6.240 using amino acids and iterated 1000 times [50]. In order to maximize the information available for phylogenetic analysis of these closely related strains, the alignments were reverse-translated to nucleotide data referencing the original CDS file with RevTrans Version 2.0 before trimming and gap removal [51]. Using codon alignments allowed us to retain the information in the more rapidly evolving third codon position. Although *Candida/Clavispora* yeasts belong to a larger lineage that recode CUG from leucine to serine, we chose to generate protein sequences using a standard codon table since we reverse-translated alignments with the original CDS (nucleotide) data. The alignments were then trimmed using the ‘gappyout’ parameter in trimAl [52]. Trimmed alignments are available at https://doi.org/10.5281/zenodo.8408925.

### Phylogenetic inference

We estimated the phylogenetic relationships among the samples first through maximum likelihood (ML) analysis of the concatenated dataset. The trimmed CDS alignments were concatenated using catfasta2phyml.pl script (https://github.com/nylander/catfasta2phyml), and the resulting alignment consisted of 1 152 540 alignment sites and 592 724 alignment patterns representing 110 input genomes. The ML tree for the concatenated dataset was estimated with RAxML-NG using the General Time Reversible (GTR) +GAMMA model of nucleotide substitution with 100 bootstrap replicates [53] to assess support.

We next used the multilocus dataset for species tree inference under a coalescent model. Gene trees were generated for each of the 619 CDS alignments with RAxML-NG using the GTR +GAMMA model and 100 bootstrap replicates. The individual ML gene trees are available at https://zenodo.org/record/8411131. These gene trees were input to ASTRAL 5.7.1 [54] for species tree estimation. Patterns of gene tree concordance and conflict were summarized using PhyParts [55]. This package takes an input tree (here our ASTRAL species tree) and computes the proportions of genes that support or conflict with each node as well as the proportion that are uninformative with respect to the node. The cut-off value was set to 50 % bootstrap support (i.e. genes with less than 50 % support for a given node would be deemed uninformative). The results of PhyParts were visualized with the phypartspiecharts.py script (https://github.com/mossmatters/MJPythonNotebooks/blob/master/PhyParts_PieCharts.ipynb).

### Phylogenetic signal in isolation source

*D*-statistics were used to examine whether isolation source shows phylogenetic signal, i.e. whether more closely related species or strains tend to be similar in source (clinical or non-clinical). Isolation source for each strain was captured from metadata available in GenBank, SRA or CBS (S1 Appendix). Taxa were scored as ‘clinically isolated’ if the strain was isolated from any human body site (S1 Appendix). All other isolation sources, including insect, plant and environmental were scored as ‘non-clinically isolated’ (S1 Appendix). Fritz and Purvis’ *D* statistic (FPD) [56] was calculated for this binary trait in our ML tree using the ‘caper’ package version 1.0.3 in *R* [57]. The FPD statistic takes a value of one if the trait has a phylogenetically random distribution, and a value of zero if the trait has evolved under Brownian motion, whereby shared history will lead closely related taxa to have similar trait values. To assess if the distribution is random, the null distribution was created by randomly reshuffling the tip states 1000 times, and by evolving these traits on the phylogeny under a Brownian motion model 1000 times. A *D* statistic closer to one suggests that the observed binary trait has a phylogenetically random distribution across the tips (chance), whereas a value closer to zero suggests that observed trait has evolved by Brownian motion [56]. Values of *D* can fall outside of a 0–1 range when phylogenetic signal is high [56].

### Topology testing

Given that branch lengths are short for relationships within species and that isolation source does not appear to be randomly distributed (see results), we used two approaches to directly address whether the data can reject the monophyly of clinically-isolated strains of *C. auris* and *C. haemulonii*. First, we investigated if any of the 619 individual ML gene trees estimated a topology where clinical isolates form a clade compared to non-clinical isolates in each species. We created and applied filters separately for *C. auris* and *C. haemulonii* using PAUP* v. 4.0a169 [58]. Second, we carried out Kishino-Hasegawa tests [59] in a maximum likelihood framework in IQ-tree [60]. We applied the same constraints as for the filtering, repeating the tests for *C. auris* and *C. haemulonii*. We used the maximum likelihood tree from the concatenated dataset as the unconstrained tree.

## Results/Discussion

### Diversifying taxon sampling in the *Candida/Clavispora* clade

Species belonging to the *Candida/Clavispora* clade were identified through a review of existing phylogenetic estimates based on whole genome and marker gene sequence data, as well as historical taxonomic data from publicly available databases [18,26,31]. From this review, we estimated that 45 species comprise the *Candida/Clavispora* clade (S1 Appendix). Of those 45 species, however, 27 species did not have raw whole genome sequence data or isolates available (S1 Appendix). We identified four species with raw whole genome sequence data from GenBank (*Candida blattae*, *C. intermedia*, *C. oregonensis*, and *C. thailandica*) and five species with isolates available for sequencing (*Candida dosseyi*, *C. hainanesis*, *C. heveicola, C. mogii*, and *C. ruelliae*) (Table 1). These nine species were all isolated from environmental substrates and have not been, to our knowledge, previously isolated from human sources. The remaining nine *Candida/Clavispora* species in our dataset have abundant isolates or sequence data available, although the majority of these have been isolated from human sources.

**Fig. 1.**
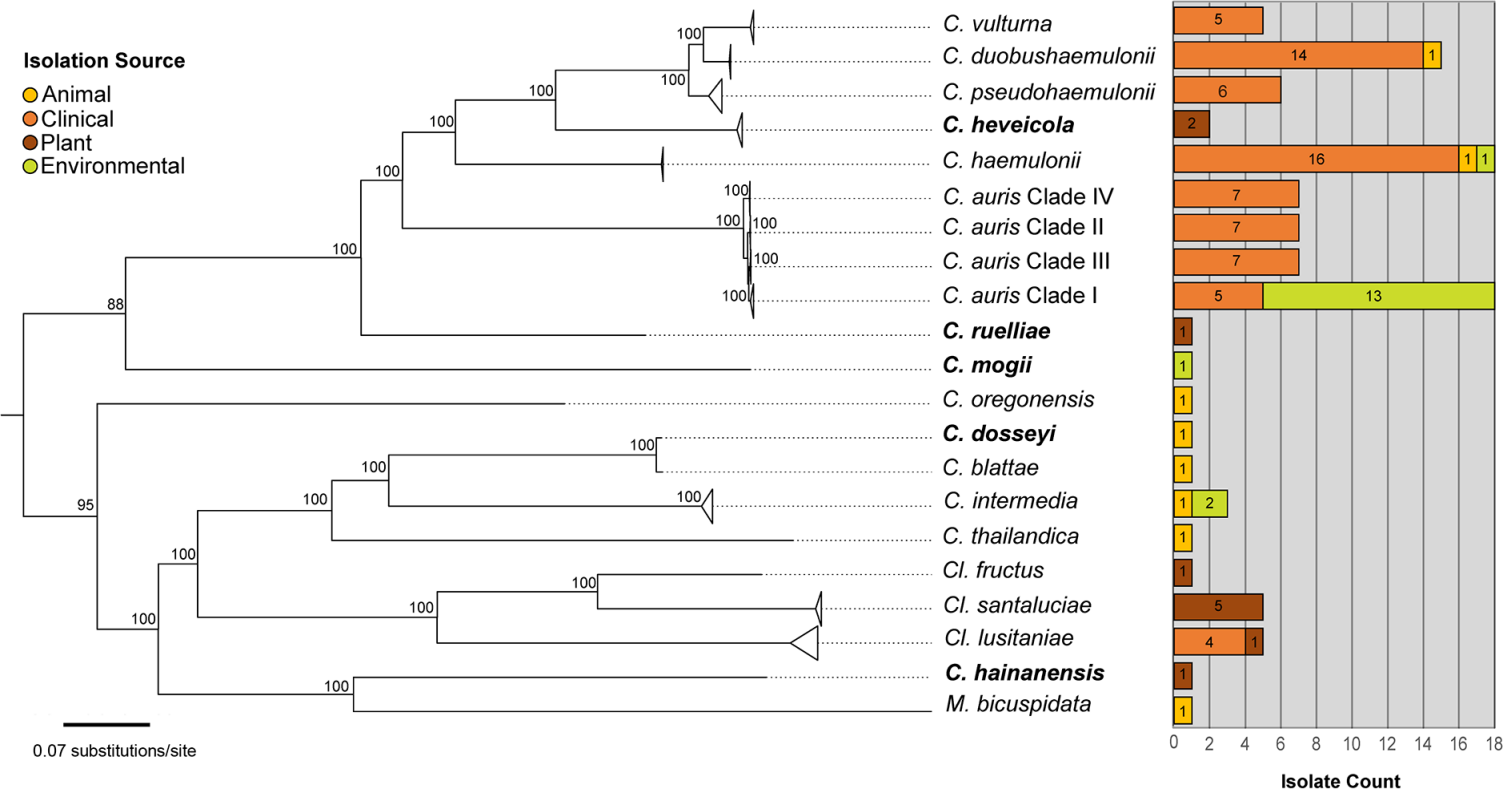
Multiple origins of pathogenic lifestyle in sampled *Candida/Clavispora* yeasts. A collapsed maximum likelihood tree rooted on *S. cerevisiae* (pruned) based on 619 orthologous genes from 108 isolates using a GTR+GAMMA model and 100 bootstrap replicates. Triangles represent a collapsed node. Bolded species are whole genomes newly sequenced as part of this study. Bar graph represents the sampling diversity of isolation sources by category used in this dataset. An expanded version of this phylogeny can be found in Fig. S1.

To diversify isolation sources within our dataset, we identified and included environmentally isolated strains from species that have also been isolated from human sources (Table 1). Although these isolation sources do not necessarily correspond to an ecological niche, they do represent conditions in which viable yeast cells were cultured and maintained. In total, our final dataset included 71 strains isolated from human sources in clinical settings and 37 strains isolated from plants, non-human animals, or other environments (S1 Appendix, Fig. 1). We selected *Metschnikowia bicuspidata* (also in *Metschnikowiaceae*) and the more distantly related *Saccharomyces cerevisiae* as outgroups. We rooted all trees on *S. cerevisiae*.

**Fig. 2.**
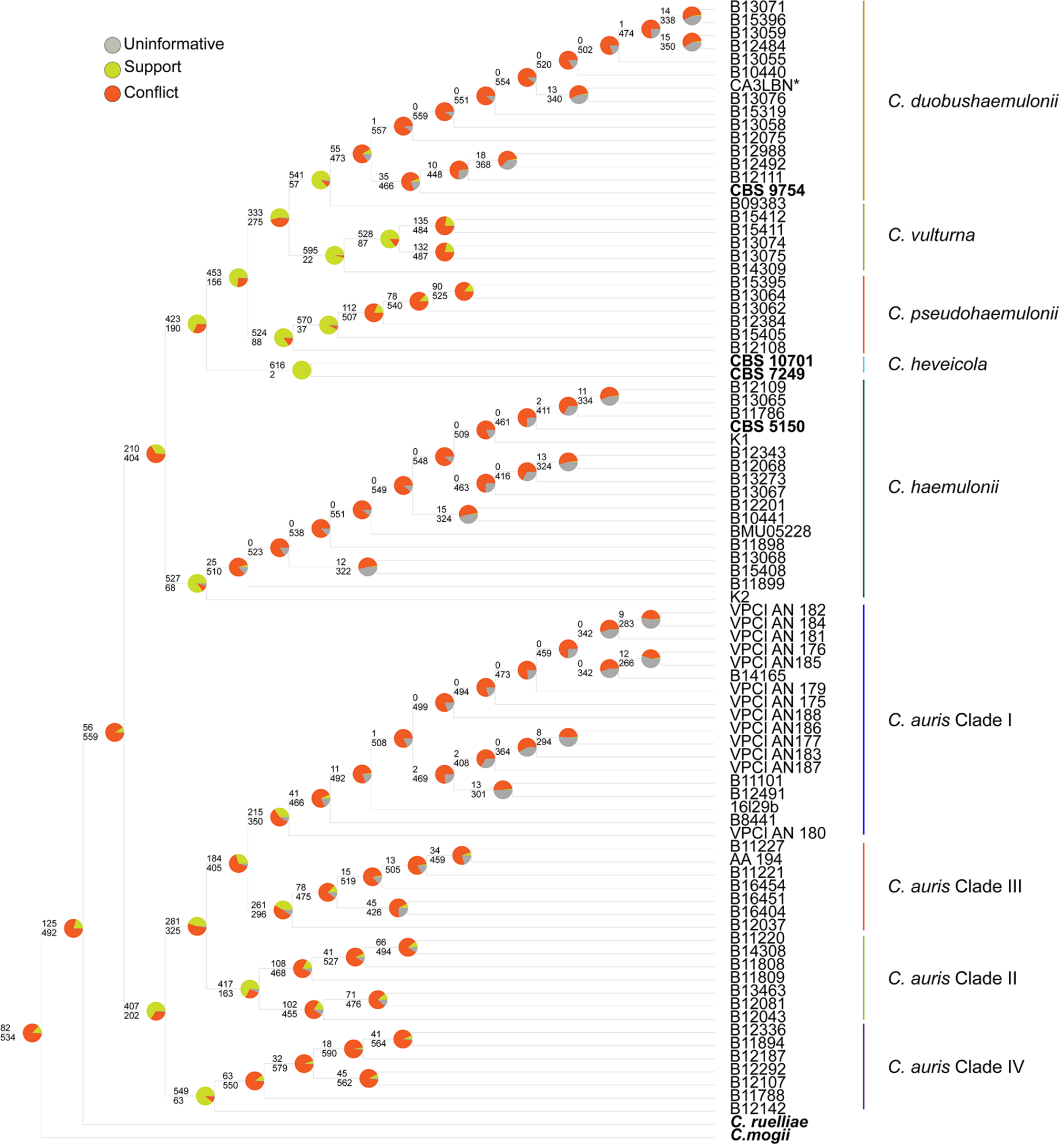
Recent divergences of *C. auris* and *C. haemulonii* show conflict among orthologous ML genes trees. A coalescent species tree estimation based on 619 ML gene trees in ASTRAL. Gene conflict was assessed by a conflict analysis using PhyParts. Green portions of the pie charts show the proportion of 619 ML gene trees that support the presented topology with 50 % or higher bootstrap. Red portions of the pie charts show ML gene trees that support an alternate topology with 50 % or higher bootstrap, while the grey portions of the pie charts depict genes that are uninformative (<50 % bootstrap support).

### Reappraising phylogenomic relationships *Candida/Clavispora* clade species

From our 108 genome assemblies (82 *de novo* and 26 downloaded assemblies), we recovered 619 single-copy orthologous genes, similar to the number of shared core genes of other studies analysing broader phylogenies in this sub-Phylum [26]. ML phylogenetic inference based on the concatenated dataset showed high bootstrap support for relationships among species and clades (Fig. 1). The branch lengths separating strains were orders of magnitude shorter than those separating species, suggesting few genetic differences among the strains (Fig. S1). Given that rapid speciation events can lead to discordant gene trees [61], we also estimated a species tree using multispecies coalescent methods that account for incomplete lineage sorting [62]. The species tree topology (Fig. S1) recovered the same relationships among the species as in the ML phylogeny but differed in some of the shallow relationships among the intraspecific strains (Fig. S1). Observing the same clades corresponding to species in both analyses strongly supports the current species delimitation in the *Candida/Clavispora* clade. Moreover, gene trees were generally concordant for these species-level clades (Fig. S2). While some intraspecific clades had strong agreement across genes (e.g. the clades within *C. auris*, Fig. S2), most shallow relationships showed conflict and uncertainty across gene trees (Fig. S2), consistent with incomplete lineage sorting along short internodes [62]. Many genes are also uninformative at the intraspecific level (grey portion of pies in Fig. S2), implying that they have little phylogenetically informative variation at that scale.

#### Addition of newly sequenced yeast species broadens the evolutionary history of *Candida*/*Clavispora* clade yeasts

We recovered many previously supported relationships but also discovered new relationships, particularly for the newly sampled taxa. The addition of *C. dosseyi* [63]*, C. heveicola* [64]*, C. hainanensis* [64]*, C. mogii* [65]*,* and *C. ruelliae* [66] to the phylogenetic analyses helped resolve the uncertainty of their placement in the clade. *C. dosseyi* (isolated from an insect) is part of the *Clavispora* sub-clade of the *Candida/Clavispora* clade, sister to *C. blattae* (also isolated from an insect) (Fig. 1). These two species are nested within a larger clade with two other environmentally isolated species *Cl. intermedia* strains from sewage and wheat-straw hydrolysate, and a *C. thailandica* strain isolated from insect frass (Fig. 1). These species are sister to *Cl. lusitaniae* (Fig. 1). This topology is similar to a recent phylogenetic estimation of *Clavispora* species based on aligned proteins [28] but differs in the placement of *M. bicuspidata*, which here is sister to the newly added *C. hainanensis* (bootstrap support of 100% from the concatenated dataset; supported by 500/619 genes with 50 % or higher bootstrap, Figs S1 and S2).

*C. ruelliae,* isolated from flowers in India, is a yeast that demonstrates thermotolerance and the ability to form pseudohyphae, traits commonly associated with pathogenic potential [66]. *C. ruelliae* had been previously estimated to be part of the *C. haemulonii* complex species [31]. Prior to this study, a whole genome sequence had not been produced for the only known *C. ruelliae* isolate. In our topology, *C. ruelliae* is sister to both *C. auris* and the *C. haemulonii* complex species with 100 % bootstrap in the concatenated analysis and 125 gene trees individually in support of this topology (Figs1  2). *C. mogii*, isolated from Fucho-Miso soybean paste in 1962 [65], appears to be sister to the *C. auris*, *C. haemulonii* complex species and *C. ruelliae* clade (Figs.1  2). Although this isolate was previously estimated to be distant from these yeasts based on marker gene sequencing data [30], the topology inferred here shows 88 % bootstrap support and 82 gene trees in agreement with its placement.

#### *C.haemulonii* complex species are not exclusively human-associated yeasts

The *C. haemulonii* complex species are often presented as a single clade of multi-drug resistant, human-pathogenic fungi sister to *C. auris*, an informal taxonomic designation based on the genetic similarity of species relative to *C. haemulonii sensu stricto* [31,67, 68]. This species complex typically includes *C. haemulonii*, *C. haemulonii* var. *vulnera* (isolates K1 and K2) [68], *C. duobushaemulonii,* and *C. vulturna* which are all sister to *C. pseudohaemulonii* and *C. auris* [18,69]. To reappraise relationships within this species complex, we included representatives of each of these species, including environmentally isolated strains when available, as well as strains of environmentally isolated *Candida heveicola* [64]. To date, *C. heveicola* is the only species within this complex that has never been isolated from a human source. In contrast to previous phylogenies, we recover a topology in which *C. vulturna, C. duobushaemulonii, C. pseudohaemulonii* and *C. heveicola* form a clade sister to *C. haemulonii* and *C. haemulonii* var. *vulnera* (Fig. 1). There is high bootstrap support (100 %) and 98 % of the individual gene trees support this placement (Figs 1 and S1). Additionally, we estimate that *C. haemulonii* var. *vulnera* isolates K1 and K2 do not form a clade sister to *C. haemulonii* strains, suggesting that they are not a species variant as previously described in 1993, but are members of *C. haemulonii* [67] (Fig. 2a). This topology is likely different given that previous estimations have been based on ITS sequence data [18], isoenzymes relatedness [67], and orthologous genes from taxa that are too closely related [68]. This finding may have implications for concerns regarding the clinical misidentification of *C. haemulonii* var. *vulnera* with *C. haemulonii* based limitations of certain clinical laboratory detection methods and may warrant reevaluation of taxonomy as it relates to clinical management.

### Phylogenetic signal implicates transitions from environmental to clinical settings are clustered in some clades

We hypothesized that if humans become colonized through repeated interactions with yeasts in the natural environment, we would see clinically isolated strains interspersed within environmentally isolated strains, rather than forming a grade or a phylogenetically distinct clade. Indeed, by adding environmentally isolated strains to our sample, we find that they are commonly interspersed within clinically isolated strains in our ML phylogeny (Fig. S1). Focusing on *C. auris* and *C. haemulonii*, we were interested to know if there was any shared ancestry that might support a single divergence event between clinical and non-clinical strains within a species, especially given the high gene tree conflict observed towards the tips of our coalescence analysis (Fig. S2). However, after filtering our gene trees with a topological constraint, we did not recover any ML topologies that support environmentally isolated strains forming a distinct clade to clinical strains in either species. This suggests that there is no evidence of divergence between strains based on isolation source at the individual gene level in our sample.

We then used two statistical approaches to test how these patterns relate to our hypotheses in *C. auris* and *C. haemulonii*: Fritz and Purvis *D* (FPD) statistic and topology testing. Given the large number of taxa used in our ML analysis (*n*=108) and the prevalence of clinically isolated strains in the dataset, we measured the strength of phylogenetic signal with FPD, which is based on the sum of sister-clade differences in an ML phylogeny. Scoring isolation source as a binary trait (clinical vs. non-clinical), we estimated *D* to be −0.0213, which suggests that the patterns recovered in our ML topology are less likely due to chance alone (*p value*=0) and more likely to have evolved following BM (*p value*=0.523). Therefore, closely related taxa are more likely to exhibit the same state due to their shared ancestry and some lineages may be more likely to live in human environments. For example, clinically isolated *C. haemulonii* strains appear clustered on the phylogeny, even though they are sourced from different geographic locations (Fig. 2a). The strength of this signal (*D=*−0.0213) shows that patterns of isolation source as a binary trait (clinical vs. non-clinical) are likely based on evolved continuous traits within these yeasts and that it is not phylogenetically random [56]. This suggests that some lineages are more likely to be isolated from human sources, which is important to consider given that global surveillance of *Candida/Clavispora* species is often not focused on non-*auris* species.

Even though clinical strains show some clustering on the phylogeny, our data strongly reject a single transition from the natural environment into clinical settings. To statistically evaluate the presence of clinically isolated strains forming clades, we compared the log-likelihood scores of the more likely ML topology estimated without constraints to a topology where environmental isolates were constrained to a monophyletic group. We applied a KH test to constrained and unconstrained topologies of *C. auris* and *C. haemulonii*, given that both had more than one environmentally isolated strain available and demonstrated the FPD clumping pattern described above. In both topology tests, the constrained topology performed significantly worse (lower log likelihood) than the unconstrained topology (Table 2), demonstrating that the data can reject the clinical monophyly hypothesis.

**Table 2.** Summary of the one-sided Kishino-Hasegawa (KH) topology test for environmentally constrained topologies of *C. auris* and *C. haemulonii* compared to the unconstrained ML topology

	ML topology (unconstrained), logL	*C. auris* environmentally constrained, logL	*C. haemulonii* environmentally constrained, logL
Log likelihood	−14370660.53	−14388686.15	−14370573.87
*p* value of one-sided Kishino-Hasegawa test	1	<0.0001	<0.0001

For both statistical tests, it is possible that the addition of newly identified, non-clinically isolated strains of these species could clarify the topology by way of non-clinical sister groups or even a grade of non-clinical samples, which could support a transition from the natural environment to human, but even so, there would still be non-clinical strains nested within clinical isolates, consistent with escapes from human habitats or multiple environmental spillovers into human populations. Although it would be challenging to achieve sufficiently dense sampling to robustly estimate the number and direction of transitions, the intermixing of clinical and non-clinical isolates in our analyses highlights the breadth of environments these yeasts inhabit and suggests flexibility in their growth conditions. It also implies that at least some lineages are more likely to be able to live in human environments, in which case, there are likely intrinsic factors that underly this flexibility.

### Chronicling patterns of human association using environmentally isolated *C. auris* strains

With our whole genome data, we estimated relationships between the newly identified environmental *C. auris* strains and clinical strains representing the four known sub-clades of *C. auris* (Fig. 2). The isolation of *C. auris* from salt marshes and sandy beaches in the Nicobar and Andaman Islands marked the first time this species had been found in the natural environment, suggesting a possible environmental niche for the deadly pathogen which bolstered previous hypotheses about its ecological origins in hot, humid climates [3,6]. Similar to previous analyses, we recovered four, highly supported sub-clades of *C. auris*, which together form a clade sister to the *C. haemulonii* species complex [10,18] (Fig. 2a; S1 Appendix). The branch subtending *C. auris*, is relatively long with many substitutions over time (Fig. 1), while branches within the subclades have little agreement across gene trees (Fig. S2) and are very short, consistent with previous intraspecific analyses of *C. auris* [10,18].

**Fig. 3.**
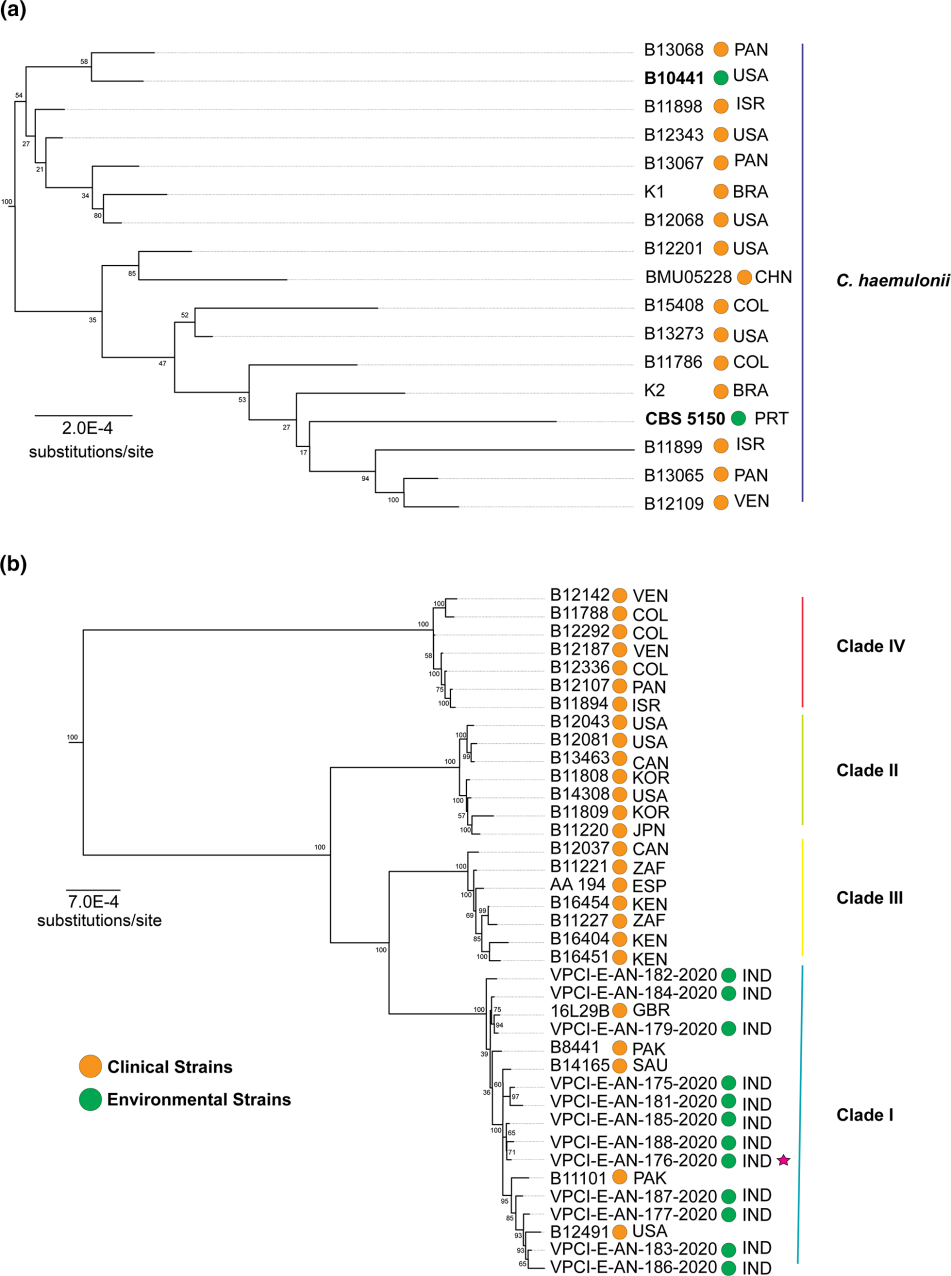
Strains isolated from non-clinical sources are not separate environmental lineages of *C. haemulonii* and *C. auris*. Maximum likelihood tree rooted on *S. cerevisiae* based on 619 orthologous genes using a GTR+GAMMA model and 100 bootstrap replicates. Three letters represent the country where the sample was collected. (**a**) Zoomed in phylogeny showing sampled *C. haemulonii* strains. Green circles represent environmentally isolated strains. CBS 5150 was isolated from seawater in Portugal in 1972 and B10441 was isolated from blue-striped grunt in 1962. (**b**) Zoomed in phylogeny showing sampled *C. auris* strains. Corresponding clade number was assigned based on NCBI designation. Green circles represent environmentally isolated strains collected by [6] in the South Andaman Islands. VPCI-E-AN-180 was pruned from the tree due to poor sequence quality. The red star denotes an antifungal susceptible, environmentally isolated *C. auris* strain (fluconazole MIC, 8 mg l^−1^; amphotericin B MIC, 1 mg l^−1^) [29].

Although we recovered a similar topology to Arora *et al*. [6] when these environmentally isolated strains from the Andaman Islands are included in the phylogeny, we disagree with their interpretation of the relationships recovered in their phylogeny. Based on our data we found no genetic basis for a diverged lineage of ‘wild’ *C. auris* strains, as the environmentally isolated strains from salt marshes were interspersed within the clinically isolated *C. auris* strains (Fig. 3b). Given that *C. auris* and relatives can survive harsh environments, such as seawater, it is more likely that these relationships likely represent a seeding of the natural environment from human sources and not a diverged lineage resembling the ancestral state of *C. auris*. Additionally, the isolate VPCI-E-AN 176–2020 that was described as susceptible to fluconazole (8 mg l^−1^) and amphotericin B (1 mg l^−1^) and a slow grower at 42 °C, was not genetically distinct from other environmentally and clinically isolated strains and is therefore unlikely a ‘wild’ strain of *C. auris* that had yet to evolve thermotolerance. In contrast, this strain could have just as easily lost the ability to grow above 45 °C, suggesting that thermotolerance is a trait that has evolved because of adaptation to the human body, rather than an adaptation to warming ambient temperatures. Even though thermotolerance is a critical trait for fungi to survive in the human body [12], the expansion in the distribution of these yeasts outside tropical regions challenges the hypothesis that global warming is a major evolutionary driver of pathogenicity in this clade [3,6]. Additional evidence is needed to understand how selection on thermotolerant traits operates in yeasts from areas where the effects of global warming are less pronounced (average ambient temperature <37 °C) and areas where these effects raise average ambient temperatures above 37 °C.

Although we did not find evidence that these environmentally isolated strains represent a separate lineage from the natural environment; our findings may provide additional context on the outstanding question of geographic clustering of *C. auris* strains. A recent study scanning metagenomic databases recovered marker-gene traces of *C. auris* and relatives in an even broader geographic range in samples from diverse environments, such as amphibian skin and soil [20,70]. These findings build on the presence of *C. auris* in marine environments [6,19]. Taken together, this evidence suggests that *C. auris* strains have been moving between humans and the environment much longer than expected. Indeed, the detection of *C. auris* in wastewater could represent a conduit for moving yeast populations between humans and the environment [20,21]. It is therefore plausible the five distinct clades recovered in phylogenetic analysis could just as easily represent five ongoing outbreak lineages that have undergone genetic changes in the human body rather than five unique environmental lineages from different geographic origins.

## Conclusion

The patterns of phylogenetic relationships recovered in our analyses suggest many transitions between humans and other environments in *C. auris* and close relatives. Using isolation source as a proxy for ecological breadth, we found that yeasts in the *Candida/Clavispora* clade likely have a high capacity for transition between many different environments. Specifically, we found that environmental isolates are commonly nested within clades of clinical isolates, as opposed to forming distinct clades sister to clinically isolated strains or forming a grade in which clinical isolates are nested. This challenges the hypothesis that there are naïve lineages living in the natural environment that are genetically distinct from lineages circulating amongst humans. In addition to regulatory and coding sequence changes, these traits may be tied to changes in gene content, such as gene gain and loss as is the case for *C. albicans* [71], which we can now begin to explore with the growing body of genome assemblies.

In these *Candida/Clavispora* species as in other *Saccharomycotina* species, colonization of a human host could be a side effect of their physiological capability to survive in diverse, harsh conditions and not necessarily the gain of specialized virulence traits, which likely evolve due to selection pressure present during infection [23]. This could represent a longer co-evolutionary history with humans than previously thought, which supports the hypothesis that the progression to serious disease is secondary to increases in immunocompromised individuals in the population. A broad set of *Candida/Clavispora* samples detected with marker-based sequencing from diverse environments across the globe reinforces the idea that, likely, immunocompromised or sick individuals are frequently encountering these yeasts in the natural environment, and potentially contributing adapted strains to environmental reservoirs [3,19,21]. The recent detection of these yeasts in humans might correspond with increases in at-risk populations over the past 30 years, for example, changes in cancer, transplant, and HIV patient populations, ultimately representing a change in host environment [34]. This explanation fits logically with what is known about the transmission dynamics of these yeasts in hospital settings, specifically their ability to exclusively cause severe disease or long-term asymptomatic carriage in heavily medicated (antibiotics, antifungal, immunosuppressants) patient populations with indwelling medical devices [72].

As we continue to see an increase in vulnerable patient populations, evidenced by recent increases in intensive-care hospitalization due to COVID-19 [4], we should be vigilant in our surveillance for yeasts belonging to this clade being isolated clinically from humans, and work to better understand the human-fungal interactions in these populations. Increased environmental sampling and obtaining isolates from these diverse sources will greatly enhance the phylogenomic dataset generated in this study and help to form a better picture of the evolutionary dynamics of the human pathogenic lifestyle in this clade.

## supplementary material

10.1099/mgen.0.001233 Uncited Supplementary Material 1.

## Abbreviations

FPD: Fritz and Purvis’ D statistic
ML: maximum likelihood
