# Supplementary material for: Patterns recovered in phylogenomic analysis of Candida auris and close relatives implicate broad environmental flexibility in Candida/Clavispora clade yeasts
Source: Microb Genom. 2024 Apr 17;10(4):001233. doi: 10.1099/mgen.0.001233 (PMC11092196; doi:10.1099/mgen.0.001233)
Supplement: Uncited Supplementary Material 1. [file mgen-10-01233-s001.pdf]

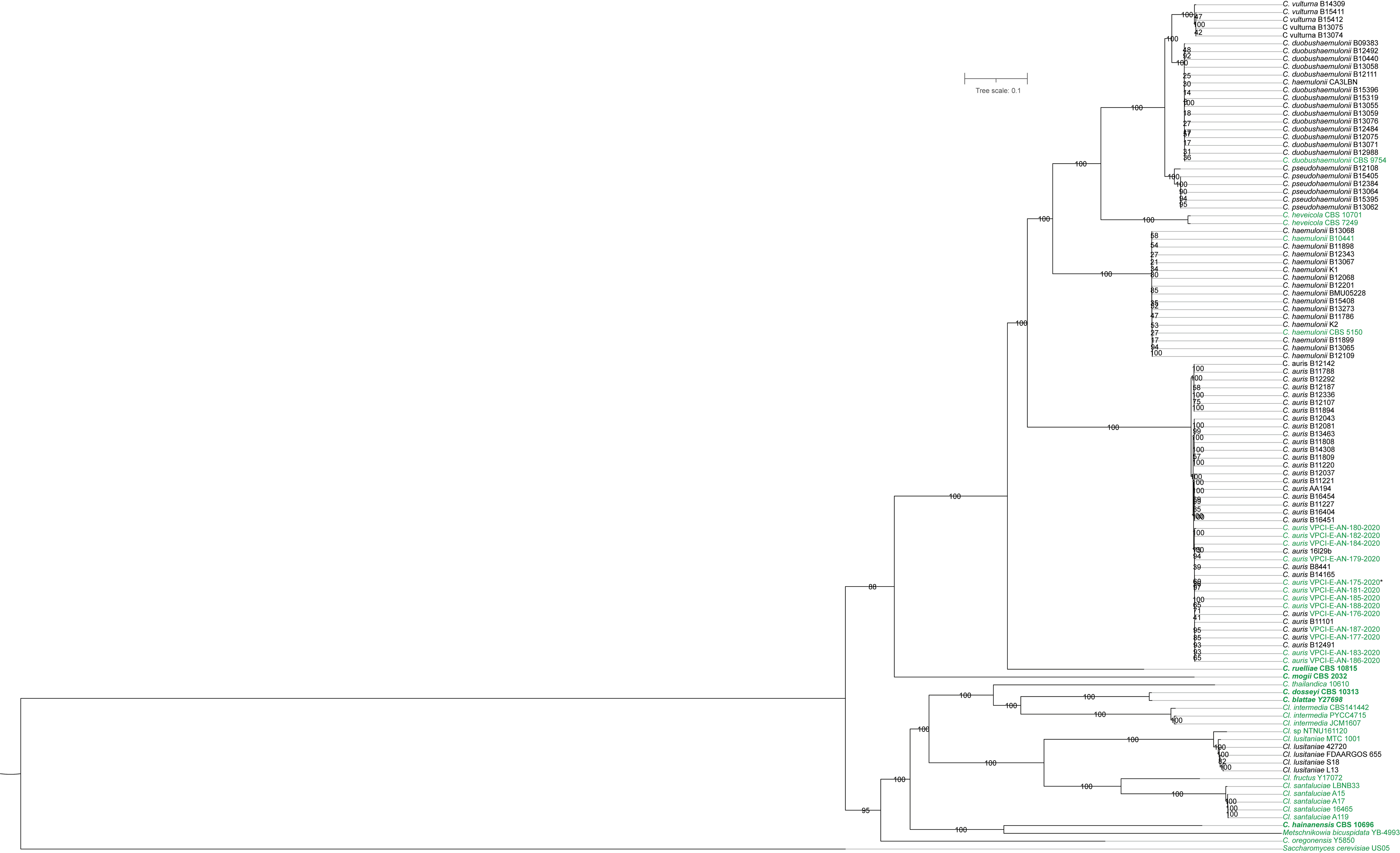
Tree scale: 0.1
C. vulturna B14309
C. vulturna B15411
C vulturna B15412
C vulturna B13075
C vulturna B13074
C. duobushaemulonii B09383
C. duobushaemulonii B12492
C. duobushaemulonii B10440
C. duobushaemulonii B13058
C. duobushaemulonii B12111
C. haemulonii CA3LBN
C. duobushaemulonii B15396
C. duobushaemulonii B15319
C. duobushaemulonii B13055
C. duobushaemulonii B13059
C. duobushaemulonii B13076
C. duobushaemulonii B12484
C. duobushaemulonii B12075
C. duobushaemulonii B13071
C. duobushaemulonii B12988
C. duobushaemulonii CBS 9754
C. pseudohaemulonii B12108
C. pseudohaemulonii B15405
C. pseudohaemulonii B12384
C. pseudohaemulonii B13064
C. pseudohaemulonii B15395
C. pseudohaemulonii B13062
C. heveicola CBS 10701
C. heveicola CBS 7249
C. haemulonii B13068
C. haemulonii B10441
C. haemulonii B11898
C. haemulonii B12343
C. haemulonii B13067
C. haemulonii K1
C. haemulonii B12068
C. haemulonii B12201
C. haemulonii BMU05228
C. haemulonii B15408
C. haemulonii B13273
C. haemulonii B11786
C. haemulonii K2
C. haemulonii CBS 5150
C. haemulonii B11899
C. haemulonii B13065
C. haemulonii B12109
C. auris B12142
C. auris B11788
C. auris B12292
C. auris B12187
C. auris B12336
C. auris B12107
C. auris B11894
C. auris B12043
C. auris B12081
C. auris B13463
C. auris B11808
C. auris B14308
C. auris B11809
C. auris B11220
C. auris B12037
C. auris B11221
C. auris AA194
C. auris B16454
C. auris B11227
C. auris B16404
C. auris B16451
C. auris VPCI-E-AN-180-2020
C. auris VPCI-E-AN-182-2020
C. auris VPCI-E-AN-184-2020
C. auris 16I29b
C. auris VPCI-E-AN-179-2020
C. auris B8441
C. auris B14165
C. auris VPCI-E-AN-175-2020*
C. auris VPCI-E-AN-181-2020
C. auris VPCI-E-AN-185-2020
C. auris VPCI-E-AN-188-2020
C. auris VPCI-E-AN-176-2020
C. auris B11101
C. auris VPCI-E-AN-187-2020
C. auris VPCI-E-AN-177-2020
C. auris B12491
C. auris VPCI-E-AN-183-2020
C. auris VPCI-E-AN-186-2020
C. ruelliae CBS 10815
C. mogii CBS 2032
C. thailandica 10610
C. dosseyi CBS 10313
C. blattae Y27698
Cl. intermedia CBS141442
Cl. intermedia PYCC4715
Cl. intermedia JCM1607
Cl. sp NTNU161120
Cl. lusitaniae MTC 1001
Cl. lusitaniae 42720
Cl. lusitaniae FDAARGOS 655
Cl. lusitaniae S18
Cl. lusitaniae L13
Cl. fructus Y17072
Cl. santaluciae LBNB33
Cl. santaluciae A15
Cl. santaluciae A17
Cl. santaluciae 16465
Cl. santaluciae A119
C. hainanensis CBS 10696
Metschnikowia bicuspidata YB-4993
C. oregonensis Y5850
Saccharomyces cerevisiae US05

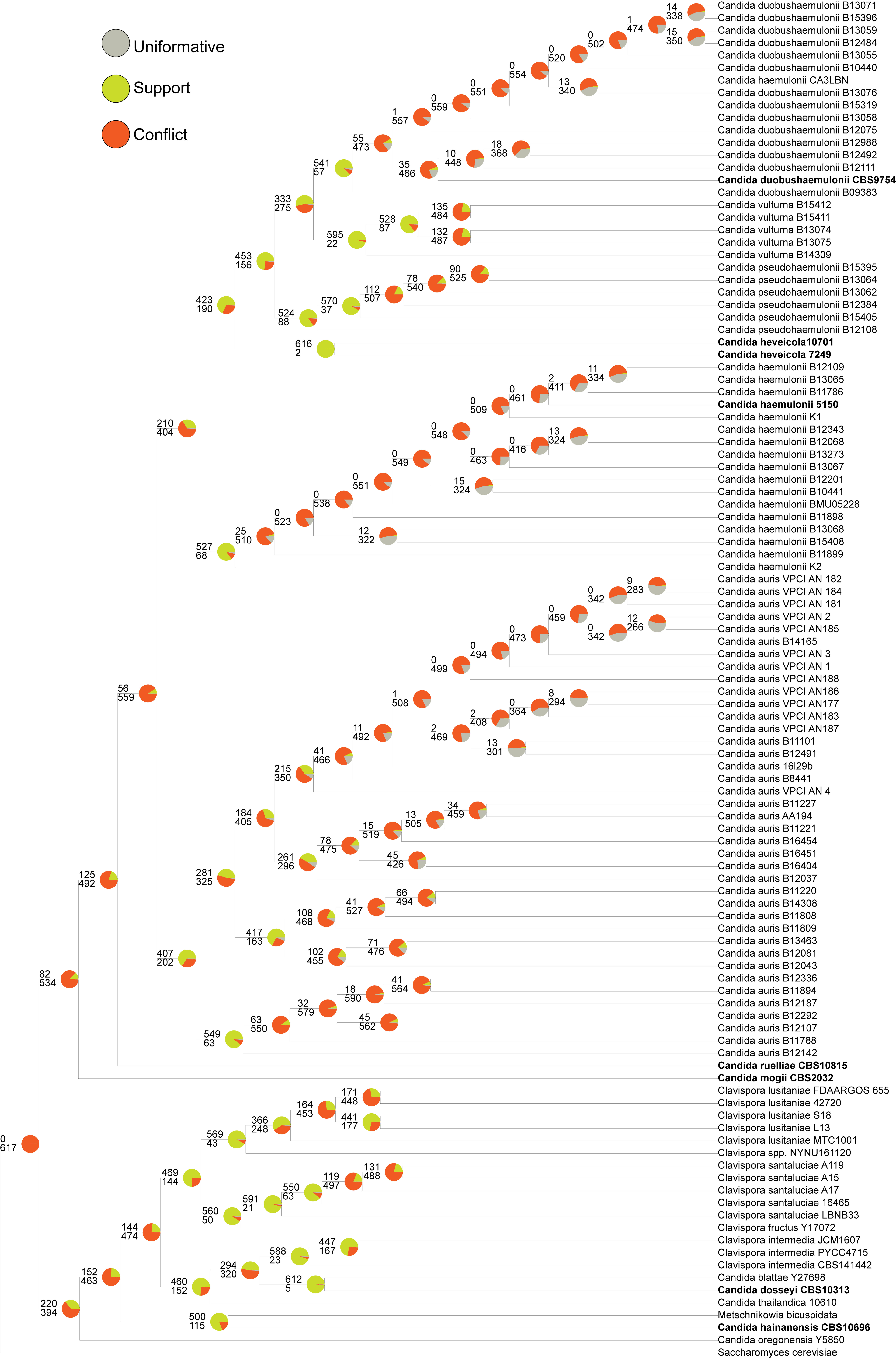

Uniformative
Support
Conflict
Candida duobushaemulonii B13071
Candida duobushaemulonii B15396
Candida duobushaemulonii B13059
Candida duobushaemulonii B12484
Candida duobushaemulonii B13055
Candida duobushaemulonii B10440
Candida haemulonii CA3LBN
Candida duobushaemulonii B13076
Candida duobushaemulonii B15319
Candida duobushaemulonii B13058
Candida duobushaemulonii B12075
Candida duobushaemulonii B12988
Candida duobushaemulonii B12492
Candida duobushaemulonii B12111
Candida duobushaemulonii CBS9754
Candida duobushaemulonii B09383
Candida vulturna B15412
Candida vulturna B15411
Candida vulturna B13074
Candida vulturna B13075
Candida vulturna B14309
Candida pseudohaemulonii B15395
Candida pseudohaemulonii B13064
Candida pseudohaemulonii B13062
Candida pseudohaemulonii B12384
Candida pseudohaemulonii B15405
Candida pseudohaemulonii B12108
Candida heveicola10701
Candida heveicola 7249
Candida haemulonii B12109
Candida haemulonii B13065
Candida haemulonii B11786
Candida haemulonii 5150
Candida haemulonii K1
Candida haemulonii B12343
Candida haemulonii B12068
Candida haemulonii B13273
Candida haemulonii B13067
Candida haemulonii B12201
Candida haemulonii B10441
Candida haemulonii BMU05228
Candida haemulonii B11898
Candida haemulonii B13068
Candida haemulonii B15408
Candida haemulonii B11899
Candida haemulonii K2
Candida auris VPCI AN 182
Candida auris VPCI AN 184
Candida auris VPCI AN 181
Candida auris VPCI AN 2
Candida auris VPCI AN185
Candida auris B14165
Candida auris VPCI AN 3
Candida auris VPCI AN 1
Candida auris VPCI AN188
Candida auris VPCI AN186
Candida auris VPCI AN177
Candida auris VPCI AN183
Candida auris VPCI AN187
Candida auris B11101
Candida auris B12491
Candida auris 16l29b
Candida auris B8441
Candida auris VPCI AN 4
Candida auris B11227
Candida auris AA194
Candida auris B11221
Candida auris B16454
Candida auris B16451
Candida auris B16404
Candida auris B12037
Candida auris B11220
Candida auris B14308
Candida auris B11808
Candida auris B11809
Candida auris B13463
Candida auris B12081
Candida auris B12043
Candida auris B12336
Candida auris B11894
Candida auris B12187
Candida auris B12292
Candida auris B12107
Candida auris B11788
Candida auris B12142
Candida ruelliae CBS10815
Candida mogii CBS2032
Clavispora lusitaniae FDAARGOS 655
Clavispora lusitaniae 42720
Clavispora lusitaniae S18
Clavispora lusitaniae L13
Clavispora lusitaniae MTC1001
Clavispora spp. NYNU161120
Clavispora santaluciae A119
Clavispora santaluciae A15
Clavispora santaluciae A17
Clavispora santaluciae 16465
Clavispora santaluciae LBNB33
Clavispora fructus Y17072
Clavispora intermedia JCM1607
Clavispora intermedia PYCC4715
Clavispora intermedia CBS141442
Candida blattae Y27698
Candida dosseyi CBS10313
Candida thailandica 10610
Metschnikowia bicuspidata
Candida hainanensis CBS10696
Candida oregonensis Y5850
Saccharomyces cerevisiae

SRA Accession	Species	Strain	C.auris Clade	Geographic Location	Substrate	Source Descriptor	Collect Date	CDS	Reference
SRR13303958	C. auris	VCPI-E-AN-188-2020	1	India	Salt marsh	Envrionmental	2021	5597	Arora et al. 2021
SRR13303959	C. auris	VCPI-E-AN-187-2020	1	India	Salt marsh	Envrionmental	2021	5067	Arora et al. 2021
SRR13303960	C. auris	VCPI-E-AN-186-2020	1	India	Salt marsh	Envrionmental	2021	5048	Arora et al. 2021
SRR13303961	C. auris	VCPI-E-AN-185-2020	1	India	Salt marsh	Envrionmental	2021	5097	Arora et al. 2021
SRR13303962	C. auris	VCPI-E-AN-184-2020	1	India	Salt marsh	Envrionmental	2021	5205	Arora et al. 2021
SRR13303963	C. auris	VCPI-E-AN-183-2020	1	India	Salt marsh	Envrionmental	2021	5023	Arora et al. 2021
SRR13303964	C. auris	VCPI-E-AN-182-2020	1	India	Salt marsh	Envrionmental	2021	7811	Arora et al. 2021
SRR13303965	C. auris	VCPI-E-AN-181-2020	1	India	Salt marsh	Envrionmental	2021	6493	Arora et al. 2021
SRR13303966	C. auris	VCPI-E-AN-177-2020	1	India	Salt marsh	Envrionmental	2021	5375	Arora et al. 2021
SRR13094247	C. auris	VCPI-E-AN-180-20202	1	India	Salt marsh	Envrionmental	2021	7528	Arora et al. 2021
SRR13094248	C. auris	VCPI-E-AN-179-2020	1	India	Salt marsh	Envrionmental	2021	5716	Arora et al. 2021
SRR13094249	C. auris	VCPI-E-AN-176-2020	1	India	Salt marsh	Envrionmental	2021	5369	Arora et al. 2021
SRR13094250	C. auris	VCPI-E-AN-175-2020	1	India	Salt marsh	Envrionmental	2021	6963	Arora et al. 2021
SRR10851769	C. auris	B8441	1	Pakistan	Blood	Clinical	2008	5201	Chow et al. 2018
ERR2299873	C. auris	16I29b	1	UK	Bed	Clinical	2016	5234	Chow et al. 2018
SRR10851771	C. auris	B14165	1	Saudi Arabia	Urine	Clinical	2018	5103	Chow et al. 2018
SRR10461149	C. auris	B12491	1	United States	BAL	Clinical	2017	5032	Chow et al. 2018
SRR3883460	C. auris	B11101	1	Pakistan	Blood	Clinical	2014	5061	Chow et al. 2018
SRR10461159	C. auris	B13463	2	Canada	Ear fluid	Clinical	2014	5092	Chow et al. 2018
SRR3883452	C. auris	B11220	2	Japan	Auditory canal	Clinical	2005	5161	Chow et al. 2018
SRR10461263	C. auris	B11808	2	South Korea	Auditory canal	Clinical	2004	5869	Chow et al. 2018

SRR10461262	C. auris	B11809	2	South Korea	Auditory canal	Clinical	2004	5641	Chow et al. 2018
SRR10461147	C. auris	B14308	2	United States	Wound	Clinical	2018	5134	Chow et al. 2018
SRR7909356	C. auris	B12081	2	United States	Ear	Clinical	2016	5069	Chow et al. 2018
SRR7909185	C. auris	B12043	2	United States	Ear	Clinical	2016	5121	Chow et al. 2018
SRR10461234	C. auris	B16451	3	Kenya	Blood	Clinical	2014	5073	Chow et al. 2018
SRR10461253	C. auris	B12037	3	Canada	Ear fluid	Clinical	2012	5172	Chow et al. 2018
SRR10461146	C. auris	B16404	3	Kenya	Urine	Clinical	2017	5079	Chow et al. 2018
SRR10461233	C. auris	B16454	3	Kenya	Vascular tip	Clinical	2014	5155	Chow et al. 2018
SRR3883453	C. auris	B11221	3	South Africa	Blood	Clinical	2012	5223	Lockhart et al. 2017
SRR3883459	C. auris	B11227	3	South Africa	Blood	Clinical	2014	5154	
SRR10461267	C. auris	AA_194	3	Spain	Blood	Clinical	2016	5231	
SRR10461194	C. auris	B12187	4	Venezuela	Blood	Clinical	2016	5215	
SRR7140028	C. auris	B12336	4	Columbia	Nares Swab	Clinical	2016	5212	Escandon 2018
SRR7140041	C. auris	B11788	4	Columbia	Blood	Clinical	2015	5143	Escandon 2018
SRR7140058	C. auris	B12292	4	Columbia	Environmental	Clinical	2016	5206	Escandon 2018
SRR10461257	C. auris	B11894	4	Israel	Blood	Clinical	2014	5173	
SRR10461178	C. auris	B12107	4	Panama	Urine	Clinical	2016	5479	
SRR10461134	C. auris	B12142	4	Venezuela	Urine	Clinical	2012	5084	
SRR23882032	C. dosseyi	CBS_10313	NA	USA	Insect gut	Animal	2005	6430	
NA	C. duobushaemulonii	B09383	NA	USA	Blood	Clinical	2011	5055	Munoz et al. 2018
SRR23882033	C. duobushaemulonii	CBS_9754	NA	Germany	Insect	Animal	2012	5299	Cendejas-Bueno et al. 2012
SRR11092034	C. duobushaemulonii	B10440	NA	USA	Foot ulcer	Clinical	1990	5100	Gade et al. 2020

SRR11091944	C. duobushaemulonii	B12075	NA	USA	Scalp	Clinical	2016	5197	Gade et al. 2020
SRR11091981	C. duobushaemulonii	B12111	NA	Venezuela	Blood	Clinical	2011	5316	Gade et al. 2020
SRR11091986	C. duobushaemulonii	B12484	NA	USA	Skin	Clinical	2017	5106	Gade et al. 2020
SRR11091975	C. duobushaemulonii	B12492	NA	USA	Ear Fluid	Clinical	2017	5162	Gade et al. 2020
SRR11091957	C. duobushaemulonii	B12988	NA	USA	Nail	Clinical	2017	5143	Gade et al. 2020
SRR11091941	C. duobushaemulonii	B13055	NA	Panama	CVC Tip	Clinical	2016	5557	Gade et al. 2020
SRR11091938	C. duobushaemulonii	B13058	NA	Panama	Toenail	Clinical	2016	5685	Gade et al. 2020
SRR11091937	C. duobushaemulonii	B13059	NA	Panama	Blood	Clinical	2016	5909	Gade et al. 2020
SRR11091998	C. duobushaemulonii	B13071	NA	Panama	Urine	Clinical	2017	5394	Gade et al. 2020
SRR11091994	C. duobushaemulonii	B13076	NA	Panama	Skin	Clinical	2017	5312	Gade et al. 2020
SRR11091945	C. duobushaemulonii	B15319	NA	USA	Bone	Clinical	2018	5249	Gade et al. 2020
SRR11091988	C. duobushaemulonii	B15396	NA	Columbia	Eye Secretion	Clinical	2017	5338	Gade et al. 2020
NA	C. haemulonii	B11899	NA	Israel	Wound	Clinical	2015	5064	Gade et al. 2020
NA	C. haemulonii	BMU05228	NA	China	Blood	Clinical	2010	5115	Liu et al. 2019
SRR11091980	C. haemulonii	B10441	NA	USA	Fish	Animal	1962	5285	Gade et al. 2020

SRR11091979	C. haemulonii	B11786	NA	Columbia	Blood	Clinical	2016	5213	Gade et al. 2020
SRR11091974	C. haemulonii	B11898	NA	Israel	Wound	Clinical	2014	5307	Gade et al. 2020
SRR11091971	C. haemulonii	B12068	NA	USA	Bronchial Wash	Clinical	2018	5343	Gade et al. 2020
SRR11091970	C. haemulonii	B12109	NA	Venezuela	Blood	Clinical	2011	8501	Gade et al. 2020
SRR11092026	C. haemulonii	B12201	NA	USA	Foot	Clinical	2016	5297	Gade et al. 2020
SRR11092025	C. haemulonii	B12343	NA	USA	Wound	Clinical	2016	5353	Gade et al. 2020
SRR11092021	C. haemulonii	B13065	NA	Panama	Vaginal Secretion	Clinical	2016	5866	Gade et al. 2020
SRR11092020	C. haemulonii	B13067	NA	Panama	Toenail	Clinical	2017	5646	Gade et al. 2020
SRR11092019	C. haemulonii	B13068	NA	Panama	Blood	Clinical	2017	5665	Gade et al. 2020
SRR11092016	C. haemulonii	B13273	NA	USA	Bone	Clinical	2017	5257	Gade et al. 2020
SRR11092005	C. haemulonii	B15408	NA	Columbia	Peritoneal Fluid	Clinical	2017	5390	Gade et al. 2020
NA	C. haemulonii	CA3LBN	NA	Lebanon	Clinical	Clinical	2020	5098	
SRR23882034	C. haemulonii	CBS_5150	NA	Portugal	Seawater	Envrionmental	2012	5265	Cendejas-Bueno et al. 2012
NA	C. haemulonii var. vulnera	K1	NA	Brazil	Blood	Clinical	2010	5160	Rodrigues et al. 2020
NA	C. haemulonii var. vulnera	K2	NA	Brazil	Blood	Clinical		5171	
SRR23882035	C. hainanensis	CBS_10696	NA	China	Flower - Magnoliaceae	Plant	2008	5195	Wang et al. 2008
SRR23882036	C. heveicola	CBS_7249	NA	China	Sap Rubber Tree	Plant	2008	8246	Wang et al. 2008
SRR23882037	C. heveicola	CBS_10701	NA	China	Sap Rubber Tree	Plant	2008	5292	Wang et al. 2008
SRR23882038	C. mogii	CBS_2032	NA	Japan	Fucho-miso	Envrionmental	1967	4737	
NA	C. pseudohaemulonii	B12108	NA	Venezuela	Blood	Clinical	2011	5007	Munoz et al. 2018
SRR11092001	C. pseudohaemulonii	B12384	NA	USA	Blood	Clinical	2016	5197	Gade et al. 2020

SRR11092000	C. pseudohaemulonii	B13062	NA	Panama	Blood	Clinical	2016	6709	Gade et al. 2020
SRR11092039	C. pseudohaemulonii	B13064	NA	Panama	Blood	Clinical	2017	5556	Gade et al. 2020
SRR11092038	C. pseudohaemulonii	B15395	NA	Columbia	Blood	Clinical	2017	5527	Gade et al. 2020
SRR11092037	C. pseudohaemulonii	B15405	NA	Columbia	Blood	Clinical	2017	5358	Gade et al. 2020
SRR23882039	C. ruelliae	CBS_10815	NA	India	Flower	Plant	2012	5210	Saluja et al. 2008
SRR11092032	C. vulturna	B13074	NA	Panama	Endothelial secretion	Clinical	2017	5750	Gade et al. 2020
SRR11091967	C. vulturna	B13075	NA	Panama	Mesh Wound	Clinical	2017	5687	Gade et al. 2020
SRR11092036	C. vulturna	B14309	NA	USA	Wound	Clinical	2018	5481	Gade et al. 2020
SRR11091966	C. vulturna	B15411	NA	Columbia	Gastric Acid	Clinical	2018	5703	Gade et al. 2020
SRR11091965	C. vulturna	B15412	NA	Columbia	Blood	Clinical	2018	5518	Gade et al. 2020
NA	Candida blattae	NRRL_Y27698	NA	Panama	cockroach gut	Animal	2002	5363	Nyugen et al. 2017
NA	Candida intermedia	jcm_1607	NA	Portugal	Sewage	Envrionmental	2014	5242	Moreno et al. 2017
NA	Candida intermedia	CBS_141442	NA	Sweden	Wheat-straw hydrolysate	Animal	2009	5285	
NA	Candida intermedia	PYCC_4715	NA	Portugal	Sewage	Envrionmental	2016	5197	
NA	Candida oregonensis	NRRL_Y5850	NA	Oregon	bark beetle frass	Animal	1962	5025	Nyugen et al. 2007
NA	Candida thailandica	CBS_10610	NA	Thailand	Insect Frass	Animal	2007	5210	Hui 2021; Jindamorakot et al. 2007
NA	Clavispora fructus	NRRL_Y17072	NA	Japan	Banana	Plant	1962	4845	Shen et al. 2018
NA	Clavispora lusitaniae	FDAARGOS_655	NA	USA	Unknown	Clinical	2020	4923	Sichtig H et al. 2019

NA	Clavispora lusitaniae	L13	NA	USA	CF Lung	Clinical	2015	4863	Demers et al. 2019
NA	Clavispora lusitaniae	S18	NA	USA	CF Lung	Clinical	2015	4776	Demers et al. 2019
NA	Clavispora lusitaniae	42720	NA	USA	Blood	Clinical	2015	4974	Demers et al. 2019
NA	Clavispora lusitaniae	MTCC_1001	NA	Israel	Citrus Peel	Plant	2017	5003	Durrens et al. 2017
NA	Clavispora santaluciae	CBS_16465	NA	Portugal	Grape	Plant	2009	4817	Franco-Duarte et al. 2022
NA	Clavispora santaluciae	a1_5	NA	Portugal	Grape	Plant	2009	4834	Franco-Duarte et al. 2022
NA	Clavispora santaluciae	a1_7	NA	Portugal	Grape	Plant	2009	4813	Franco-Duarte et al. 2022
NA	Clavispora santaluciae	a1_19	NA	Portugal	Grape	Plant	2009	4788	Franco-Duarte et al. 2022
NA	Clavispora santaluciae	LB_NB_3_3	NA	Italy	Grape	Plant	2012	4739	Bellutti et al. 2018
NA	Clavispora sp.	NYNU 161120	NA	Mexico	Aquamiel	Plant	2016	5039	Hui 2021
NA	Metschnikowia bicuspidata	YB-4993	NA	NA	NA	Na	NA	4436	
NA	Saccharomyces cerevisiae	US05	NA	NA	NA	NA	NA	5424	

Candida/Clavispora Yeasts

Candida aechmeae

Candida akabanensis

Candida asparagi

Candida aff. asparagi

Candida berkhoutiae

Candida blattae

Candida bromeliacearum

Candida carvajalis

Candida chanthaburiensis

Candida citri

Candida dosseyi
Candida ecuadorensis
Candida eppingiae
Candida ezoensis
Candida flosculorum
Candida heveicola
Candida intermedia
Candida inulinophila
Candida konsanensis
Candida kutaonensis
Candida middelhoveniana
Candida mogii
Candida oregonensis
Candida phyllophila
Candida pseudoflosculorum
Candida pseudointermedia
Candida rhizophorensis
Candida ruelliae
Candida sharkensis
Candida suratensis
Candida thailandica
Candida tolerans
Candida tsuchiyae
Candida ubatubensis
Candida vitiphila
Clavispora fructus
Clavispora opuntiae
Clavispora reshetovae
Clavispora santaluciae

Clavispora lusitaniae
Candida auris
Candida duobushaemulonis
Candida haemuloni
Candida pseudohaemulonii
Candida vulturna
